# A State Hospital for Consumptives Needed

**Published:** 1902-09

**Authors:** 


### A STATE HOSPITAL FOR CONSUMPTIVES NEEDED.

No great reduction in the mortality from tuberculosis can reasonably be expected in this State until some provision is made for the proper care of the indigent consumptives who year after year, in ever-increasing numbers, continue to crowd the public hospitals, cheap lodging houses and all other places where food and shelter are to be found. One destitute victim of tuberculosis is a greater menace to the health of a community than are ten sufferers from the same disease who are in good circumstances financially. Being made careless, and even filthy, in their habits and persons by very helplessness, and being thrown for the most part with ignorant and slovenly people who know nothing of the communicability of the disease, poor consumptives often turn the buildings in which they lodge into hot beds of infection, and may be either the direct or the indirect means of infecting an entire neighborhood. The management of these cases constitutes the greatest problem with which health officers have to deal. No "campaign of education" can possibly accomplish any good results until a suitable place for their proper care and treatment is provided. At the present time it will avail us but little to lecture poverty-stricken consumptives upon their duty to the community, or to attempt to impress those who by necessity are thrown with them with the extent of the risk they are incurring. Well-to-do cases can be easily made to understand the need for the exercise of caution, but the paupers are absolutely hopeless.

A properly planned and equipped institution established and maintained at State expense will not only provide a comfortable home for these unfortunates wherein every opportunity will be given them to recover their health, but it will secure the removal from every community of the chief disseminators of the disease. The marvelously good results obtained in Germany's sixty or more government sanatoria, and the excellent showing made by similar institutions in this country, makes plain the duty of every State and Territory, and especially that of Texas, in the matter.

The benefits to be obtained from a sanatorium of the kind referred to may be summarized as follows:

1. The public health will be protected by the removal from the cities and towns of the most dangerous disseminators of the disease to a place where they will be secure from doing further harm.

2. Outside confidence in the health and proper sanitary condition of our cities will be strengthened and made secure.

3. The institution will serve as an object lesson to show that consumption is recognized by the State as being a communicable disease. Thus many careless and ignorant individuals will be influenced to exercise caution in dealing with consumptives.

4. Cases not already hopeless will be given an opportunity to recover, and the hopeless cases will have their last days made comfortable.

5. Charitable institutions of the county and city being thus relieved of the care of consumptives, will be able to receive and care for the class of invalids for which they are intended. R.

